# Prehospital critical care dispatch: a scoping review (PHASE)

**DOI:** 10.1186/s13049-025-01450-y

**Published:** 2025-08-14

**Authors:** Peter Owen, Kim Kirby, Julian Hannah, Robert Crouch, Philip Hyde, Sarah Voss

**Affiliations:** 1 University of West of England, Bristol, United Kingdom; 2 University Hospital Southampton NHS Trust, Southampton, United Kingdom; 3 Dorset and Somerset Air Ambulance, Henstridge, United Kingdom; 4 School of Health Sciences, University of Southampton, Southampton, United Kingdom

**Keywords:** Ambulance, Critical care, Dispatch, Helicopter emergency medical services, Pre-hospital

## Abstract

**Introduction:**

Prehospital critical care (PHCC) dispatch is a vital component of emergency medical services, aiming to allocate specialised resources for critically ill or injured patients in out-of-hospital settings. This scoping review examines the existing evidence on optimising PHCC dispatch, identifies research gaps, and highlights priorities for future investigation.

**Methods:**

A systematic search of databases including CINAHL, PubMed, EMBASE, and CENTRAL from January 2004 to October 2024. We included all study types, focusing on the dispatch of PHCC assets globally.

**Results:**

The search yielded 39 studies that met the inclusion criteria. The included studies varied in design, setting and focus (e.g. Traumatic vs. Medical aetiology). Outcomes measured ranged across dispatch factors, physiological and temporal variables, with advanced intervention and survival metrics commonly used to asses dispatch effectiveness.

**Discussion:**

The review found variability in dispatch models, staffing, and outcome measures. Most studies focused on HEMS and P-HEMS, often using injury mechanisms and physiological parameters as dispatch criteria. However, their predictive accuracy is inconsistent, especially for older trauma patients. Clinician involvement improves accuracy, but the role of cognitive tools needs more study. Challenges include ethical and logistical issues in prospective studies, limited research in low- and middle-income countries, and lack of harmonised datasets for missed dispatch opportunities. Technologies like automated crash notifications and real-time video show promise but need more development validation.

**Conclusion:**

This review underscores the need for robust, prospective research to refine dispatch criteria and integrate advanced technologies. Addressing these gaps could improve resource allocation, reduce over- and under-triage, and ultimately enhance patient outcomes in PHCC systems.

**Supplementary Information:**

The online version contains supplementary material available at 10.1186/s13049-025-01450-y.

## Introduction

PHCC dispatch involves the practice and methodology of deploying specialised resources to care for the most critically ill and injured patients in out-of-hospital settings. These resources include highly skilled professionals with advanced expertise and capabilities beyond those typically available through standard EMS [1]. These specialist assets have been acknowledged for improving outcomes for this cohort of critically ill patients [2–4]. Consequently, PHCC dispatch was identified as a key priority in a recent Delphi study; three of the top 20 research topics for UK PHCC were focused on dispatch [5]. Effective dispatch is fundamental to the success of PHCC programs and services. Ambulance dispatch is inherently dynamic, open, and interconnected. Its various components often behave nonlinearly, making it a highly complex and nuanced task [6]. The limited information available during EMS calls can complicate the process of determining which patients would benefit most from the expertise of the scarce and costly PHCC resources. Efforts to refine and enhance this process, such as implementing criteria-based dispatch policies, have indicated a non-significant reduction in response times and failed to demonstrate improved mortality rates [7]. Typically, ambulances and PHCC teams are dispatched using either strict rule-based criteria, such as AMPDS (Advanced Medical Priority System) or Criteria Based Dispatch Systems, which use prompts and tend to predominate in Europe [8]. To enhance the allocation of PHCC resources, some organisations utilise specially trained personnel to manage the dispatch of specialist assets. Often stationed within the emergency call centres, these staff members screen calls to assess their suitability for PHCC team involvement. Efforts have been made to evaluate which PHCC dispatch models yield the most accurate resource allocation. For example, research into whether involving a clinician in the dispatch process adds value has produced mixed findings [9, 10]. Regardless of the method, the goal of the PHCC process is to optimise the sensitivity and specificity of tasking, ultimately reducing over and under-triage. Problems with over-triage can lead to higher financial burdens, more significant safety risks for responding PHCC assets, and the increased opportunity cost of PHCC assets not being available for severely sick or injured patients. Conversely, under-triage may lead to a lack of lifesaving interventions for those requiring it.

Phrases like “golden hour” and “platinum 10 minutes” are commonly used in EMS but often lack validity and represent arbitrary timeframes [11]. The key factor is ensuring the right team reaches the right patient to provide evidence-based interventions promptly. PHCC dispatch plays a central role in quickly identifying and assigning the appropriate specialist resources. Focusing on modifiable stages upstream and implementing improvements at these points could significantly enhance patient outcomes and benefit the broader healthcare system. However, the ideal model for PHCC dispatch remains uncertain and this scoping review aims to map the current evidence that supports these different models.

A preliminary search of MEDLINE, the Cochrane Database of Systematic Reviews, JBI Evidence Synthesis, and PROSPERO identified five existing or ongoing systematic reviews or scoping reviews into dispatch in the prehospital setting. The search uncovered one published scoping review [8] and three systematic reviews [12–14]. However, these studies differ from this scoping review in terms of design, study population, key outcomes and other aspects of their methodology. The preliminary search also identified a protocol for an ongoing systematic review listed on the PROSPERO database [15]. The protocol aims to review and analyse the literature on ambulance and helicopter response times and their impact on patient outcomes and illness severity. No scoping reviews were identified in the search that sought to map the existing empirical evidence on PHCC dispatch.

This scoping review was designed to explore the existing evidence in the literature on optimising or enhancing prehospital critical care dispatch. By doing so, we aim to pinpoint gaps in the research and highlight areas that should be prioritised for future investigation. By highlighting previous studies’ methodological strengths and weaknesses, we can build a framework for future research in this area. This scoping review aimed to identify how PHCC patients are identified in the initial call screening and what evidence currently exists to support dispatch criteria and dispatch methods. We investigated which previous methodologies have been used and what outcome measures have been reported in previous studies as measures of optimal dispatch.

## Methods

This scoping review was conducted following the PRISMA-ScR guidelines [16]. The protocol was registered on the Open Science Framework before data extraction (https://osf.io/ue5bd/).

### Data sources

We searched CINAHL, PubMed, EMBASE, and the CENTRAL trial registry using a search strategy developed with a subject specialist librarian. The finalised search strategy is provided in Supplementary File 1. The search strategy, including all identified keywords and index terms was adapted for each included database and/or information source. Studies published in any language were included. Studies published during 2004, or later were included as the team concluded that this would provide enough historical context and contain relevant sources to current practice. Non-English studies were translated using Google Translate via the automatic plugin or full text directly. Due to the large number of studies initially retrieved, the PubMed search was limited to titles and abstracts. To refine the search terms, we used the litsearchr [17] package in RStudio [18] which identified additional terms—‘Emergency Medical Dispatch,’ ‘Advanced Medical Priority Dispatch System,’ and ‘Emergency Medical Service Communication Systems’—that were not included in the original strategy. We also searched the grey literature through Google Scholar, Open Grey archive, the Bielefeld Academic Search Engine, ClinicalTrials.gov, and the medRxiv preprint server.

### Eligibility criteria

Eligibility was assessed using the Participant, Concept, Context (PCC) framework as recommended by the Joanna Briggs Institute (JBI) [19] (Table 1).

**Table 1:** Population concept context (PCC) for the scoping review

PCC Element	Definition for Review	Example
Population	Critically unwell or injured patients presenting in the prehospital setting.	Out of Hospital Cardiac Arrest (OHCA)
Concept	The scoping review will consider all studies involved in the dispatch chain (recognising, identifying, and allocating specialist resources) to care for the most critically unwell and injured patients presenting to health services.	HEMS deployment characteristics in severe Traumatic Brain Injury patients.
Context	The scoping review will focus on all systems that have a tiered response to PHCC incidents with particular attention given to the dispatching of these resources.	Helicopter Emergency Services (HEMS) dispatch

### Types of sources

This scoping review included experimental and quasi-experimental study designs, including randomised controlled trials, non-randomized controlled trials, before and after studies and interrupted time-series studies. In addition, analytical observational studies, including prospective and retrospective cohort studies, case-control studies and analytical cross-sectional studies, were also considered for inclusion. We also considered descriptive observational study designs, including case series, individual case reports and descriptive cross-sectional studies for inclusion. Qualitative studies were considered, especially those that focused on methodologies, including but not limited to designs such as phenomenology, grounded theory, ethnography, qualitative description, and action research. In addition, systematic and scoping reviews that meet the inclusion criteria were also included, depending on the research question. Finally, text and opinion papers were considered for inclusion in this scoping review.

### Study selection

**Fig. 1:**
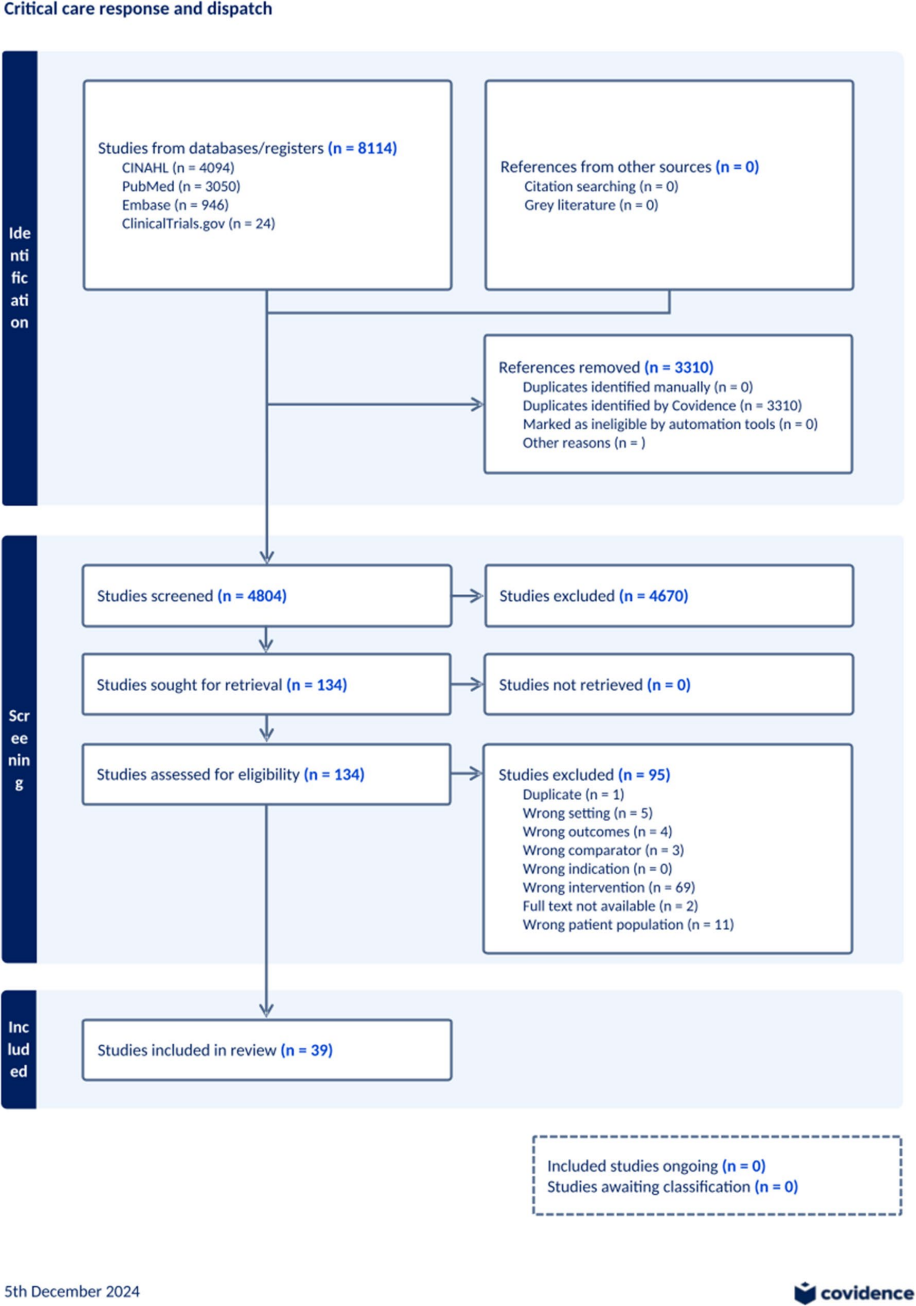
Search results and study Selection Process

Following the search, all identified citations were collated and uploaded into Covidence (https://www.covidence.org/) and PO removed duplicates. Following a pilot test, titles and abstracts were screened by PO and an independent reviewer (JH) for assessment against the inclusion criteria for the review. The full text of selected citations was then assessed in detail against the inclusion criteria by PO and one independent reviewer (JH). The reason for excluding sources of evidence at full-text review was recorded and reported in the results section (Fig. 1). There were no disagreements that could not be resolved, and the addition of a third reviewer was not required.

### Data extraction

The data collected from each study were author, year, country, study design, sample size, study duration, aetiology of the case attended (e.g. medical or trauma), primary/secondary/ composite outcomes, type of outcome measures, type of PHCC asset being dispatched, whether a clinical prediction rule was being tested, aim and study findings. Data was exported from Covidence into a word document, and PO reviewed the charting table for clarity and amendments made if required after reviewing each study in an iterative approach.

### Data analysis and synthesis

The results were described descriptively, and figures were presented using RStudio software [18].

## Results

The initial database search yielded 8114 results from four databases. The study selection process is outlined in Fig. 1. No studies were identified from other sources, likely due to the broad search criteria used initially. 3310 studies were removed as duplicates by the Covidence software. This left 4804 studies to be screened, of which 4670 were excluded based on the review of the title and abstract alone. In total, 134 studies were assessed for eligibility during a full-text review. Ninety-five studies were excluded, with wrong intervention (*n* = 69) being the most common reason for exclusion. Thirty-nine studies met the final inclusion criteria, and a summary of these, including study design, aim and outcome, is displayed in Table 2.

**Fig. 2:**
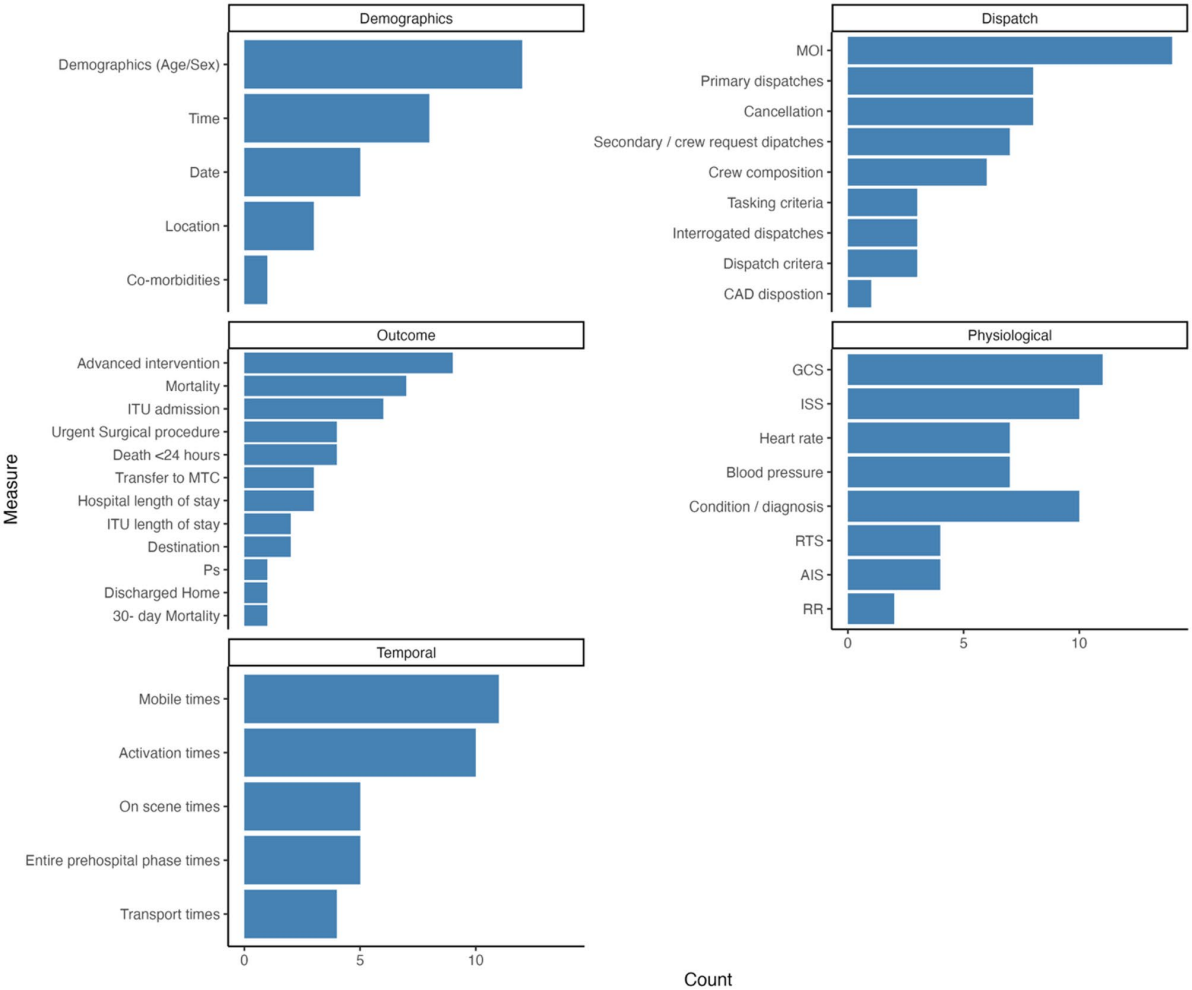
Bar chart of variables used in studies

**Fig. 3:**
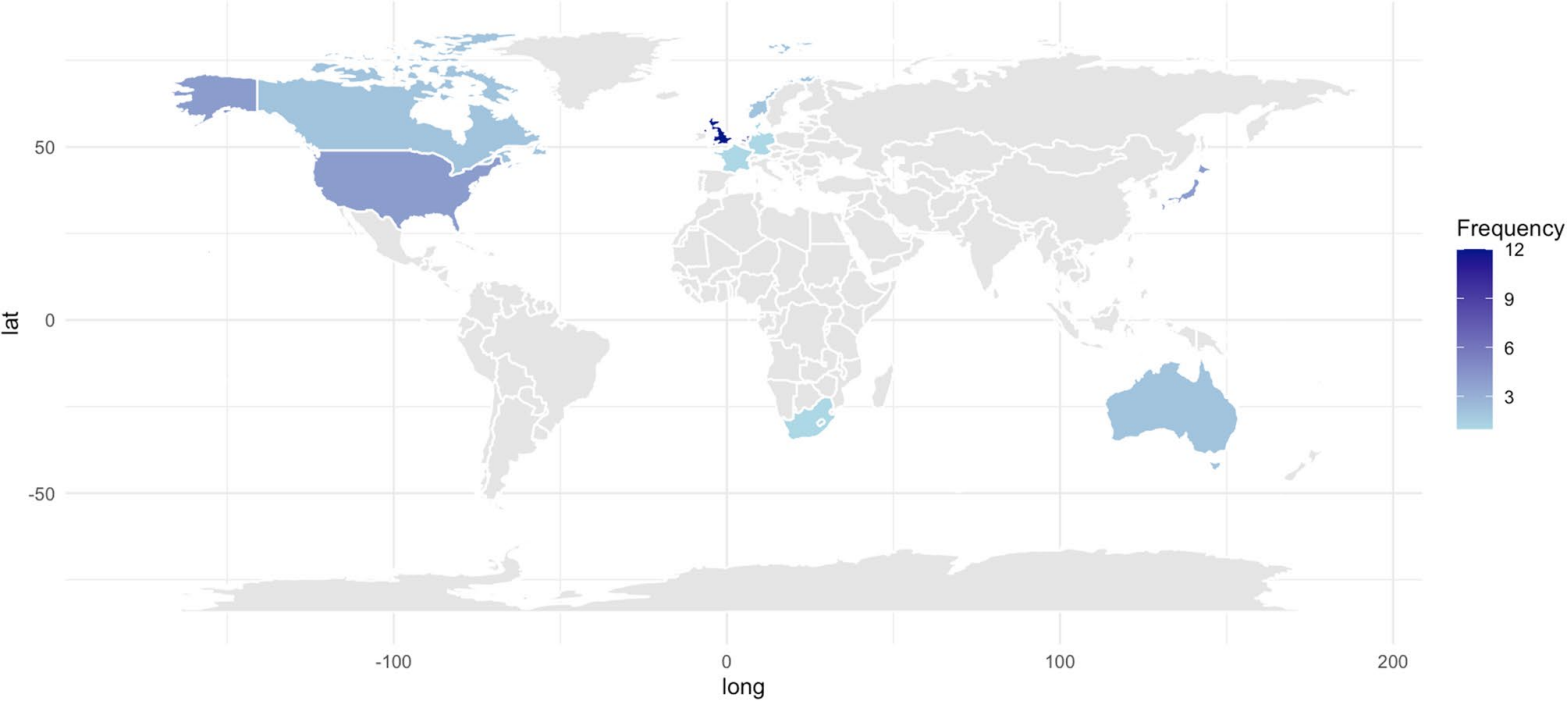
World choropleth map of country by study frequency

**Table 2:** Summary table for values

Characteristic			*N* = 39^1^
Country in which the study conducted			
Australia			2 (5.1%)
Canada			2 (5.1%)
Denmark			1 (2.6%)
France			1 (2.6%)
Germany			1 (2.6%)
Japan			4 (10%)
Netherlands			9 (23%)
Norway			2 (5.1%)
South Africa			1 (2.6%)
UK			12 (31%)
United States			4 (10%)
Publication Year			
2005			4 (10%)
2008			2 (5.1%)
2009			2 (5.1%)
2010			1 (2.6%)
2011			3 (7.7%)
2012			1 (2.6%)
2013			1 (2.6%)
2014			2 (5.1%)
2015			1 (2.6%)
2016			2 (5.1%)
2017			2 (5.1%)
2018			4 (10%)
2019			2 (5.1%)
2020			2 (5.1%)
2021			4 (10%)
2022			2 (5.1%)
2023			2 (5.1%)
2024			2 (5.1%)
Aetiology			
Medical and Trauma			18 (47%)
Respiratory			1 (2.6%)
Trauma			18(47%)
Trauma (Blunt)			1 (2.6%)
Unknown			1
Study design			
Case			1 (2.6%)
Cohort study			2 (5.1%)
Cross sectional study			2 (5.1%)
Delphi			3 (7.7%)
Feasability study			1 (2.6%)
Non—randomised experimental study			3 (7.7%)
Randomised controlled trial			1 (2.6%)
Retrospective observational			18 (46%)
Service evaluation			5 (13%)
Systematic review			3 (7.7%)
Type of PHCC asset being dispatched			
Enhanced Care Assets’			1 (2.6%)
HEMS			15 (38%)
HEMS and GEMS			1 (2.6%)
HEMS, RRV			2 (5.1%)
Mobile medical team (Physician Led)			1 (2.6%)
P—HEMS,			15 (38%)
P—HEMS, ARV			3 (7.7%)
RRV (Physician)			1 (2.6%)
Dispatch Staffing			
Automatic			2 (9.1%)
Clinician			9 (41%)
Clinician (Paramedic)			1 (4.5%)
Clinician and Non-clinician			5 (23%)
Clinician—led (Paramedic vs. Physician)			2 (9.1%)
Non-clnician			2 (9.1%)
Physician dispatch			1 (4.5%)
Unknown			17

^1^*n*(%)

### Study design, setting and aetiology

Most studies (18 of 39 (46.2%)) were retrospective observational in design [20–37]. Five (12.8%) were a service evaluation [8, 38–41] three (7.7%) were systematic reviews [7, 12, 42] and three (7.7%) were Delphi studies [43–45]. The remainder included three non-randomised experiments (7.7%), two cohort studies (5.1%), two cross-sectional studies (5.1%) [22,37], one case report (2.6%) [42] and one randomised control trial (2.6%) [47]. Twelve of the 39 studies (30.8%) were conducted in the UK [7–9, 12, 22, 30, 38, 39, 46–49] with the Netherlands conducting nine (23.1%) [20,25,28–30,33,44,45,51], and Japan [37, 50–52] and the United States [21, 31, 36, 41] both conducting four (10.3%) each. Australia [24, 25] Canada [33, 53] and Norway [35, 45] each conducted two studies (5.1%), with the remaining countries, Denmark [32] France [54] Germany [29] and South Africa [44] each conducting 1 study (2.6%). Nearly half of the selected studies (46.2%) [7,9,12,21,23,24,28,31,36,41,43,44,46,47,52,53], consisted of patients suffering from both medical and traumatic aetiologies, with a similar distribution (48.7%) [8,25–27,30,32,33–35,37−40,42,45,47,50,51,54] of studies looking at traumatic aetiologies only. One study (2.6%) looked at blunt traumatic aetiology [27] with one further study (2.6%) looking at dispatch to respiratory aetiology only [54].

### Type of asset being dispatched

HEMS [12, 21, 31, 33, 36, 39–42, 45–47, 50, 51, 53] and P- HEMS [8, 9, 20, 23–25, 27, 28, 30, 34, 35, 37, 43, 49, 52] were the most common assets being dispatched, with thirty studies between them (15 each). Studies were coded as P-HEMS (HEMS assets with a physician), only if it specifically mentioned that they included a physician in the crew mix. Similarly, studies were coded as HEMS if they did not state the specific crew mix or contained a paramedic-only crew. Only two studies (5.1%) specifically looked at ground-based responses of mobile medical teams and physician-based RRVs [52, 54]. One study (2.6%) looked at the response of “Enhanced Care Assets”, a systematic review of the literature and a term used to encompass all studies involved in a tiered response to medical and trauma incidents [7].

### Dispatch staffing and dispatch system

Twenty-two studies (56.4%) reported the staffing model used in the dispatch process. Of these, five (22.7%) used combined clinical and non-clinical staff in their dispatch [9, 22, 38, 39, 46] whilst two-thirds of studies (*n* = 13) used a clinician only [8, 20, 24–28, 30, 32, 34, 35, 48, 54] of which one study (4.5%) specified that a paramedic was used [8]. A further one study (4.5%) stated that a physician was responsible for the dispatching of their PHCC asset [54] with two studies (9.1%) comparing the dispatch between physician teams and a non-trained HEMS paramedic [24, 25]. Nine of the twenty-one studies (40.9%) did not specifically state which profession was responsible for dispatch merely stating ‘clinician’ [20, 26–28, 30, 32, 34, 35]. Non-clinicians were used in two studies (9.1%) and individuals dispatching the PHCC asset were not registered as medical professionals (Doctor/Nurse/Paramedic) but had additional training to perform the role. In two studies (9.1%) [31, 49] no staff were used in the process and ACN technology was used to dispatch PHCC assets, this was coded as automatic during the data extraction [51, 52].

Twelve studies (30.7%) [8,9,20,24,26,27,32,40,41,46,47,49] specified which dispatch system was in use and whether this was protocol-based (9/12, 75.0%) [8,9,24,26,27,32,40,47,49] or criteria-based (3/12, 25.0%) [20,41,46]. The remaining twenty-seven studies either did not mention which dispatch system was in use or, due to the study’s nature, were irrelevant to inclusion. Of the protocol-based systems, six (50.0%) [8,24,26,27,40,47] used AMPDS, and three (25%) [9,32,49] used NHS pathways. In cases where the dispatch system was not specified, the research team was able to determine if this was AMPDS or NHS pathways from the study data.

### Outcome measures and variables

Sixteen studies (41.0%) specified a primary outcome measure [8, 9, 22–29, 31, 36, 38, 49, 53, 54] which included items like transport to a major trauma centre. Nine (23.1%) specified a secondary outcome [9, 22–24, 28, 29, 49, 53, 54] such as time from the emergency call to activation of the PHCC team. Five studies (12.8%) used a composite outcome in their study design [20, 21, 36, 37, 48]. In total, 50 individual variables were used across 31 of the studies, which were recorded and then grouped into six categories for brevity (Fig. 2). These groups were dispatch-related variables, physiological, temporal, demographic, outcome and statistical variables. The outcome (13(26%)) and dispatch (11(22%)) subgroups are most frequently used as measure of success to guide dispatch.

Within the outcome subgroup, advanced intervention was the most common measure used (9(29.0%)) and related to when a PHCC-type intervention was deployed at the scene [9, 22, 23, 30, 32, 35, 37, 46, 54]. One of these studies looked at the rate of advanced intervention after arrival at the hospital in the cancelled sub-group of HEMS patients [28]. Survival metrics were used in twelve studies (38.7%), with mortality used in seven (22.5%) [20,22,35,39,40,43,53], with death < 24 h recorded in four (12.9%) [28–30,43]. ITU admission (*n* = 6) [22,28–30] and ITU length of stay (*n* = 2) [25,26] were used in nearly twenty-six per cent of the thirty-one studies.

In the dispatch subgroup, mechanism of injury (MOI) was the measure most frequently used to guide dispatch, appearing in fourteen of the thirty-one studies (45.1%) [8,9,20,21,23,26,30,37,39,40,42,50–52]. Eight studies (25.8%) used cancellation as a measure and referred to when a PHCC asset was stood down after being dispatched [9, 20, 26–29, 31, 53]. Fifteen studies (48.3%) included primary (8(25.8%)) [9,20,24,28,31,35,44,52] and secondary (7(25%)) [9,20,24,31,35,44,52] dispatches as a variable in their study design. The Interrogated dispatches variable was used in only three of the 31 studies (9.6%) [9,24,52]. Primary dispatch is when an immediate dispatch occurs based on specific criteria, with secondary dispatch occurring at the request of an attending resource on scene. Interrogated dispatch is when the agent within the dispatch centre moves outside of the protocolised questioning to ask further, more pertinent questions to guide the dispatch decision.

Dispatch arrangements were described and used as a dependent or independent variable across six of the thirty-one studies. This was when the study used the professional background of the individual making the dispatch decision as the variable being measured [8, 9, 24, 25, 48] In the case of one service evaluation used to describe individual responses for a questionnaire describing individual organisations’ dispatch arrangements [40].

Physiological data was used as a variable to assess the appropriateness of dispatch retrospectively, and there were nine variables across thirty-one studies with 55 instances where they were used. GCS was used in eleven studies (35%) [20,22,25,28,29,32,40,42,46,51,53], closely followed by the ISS used in ten (32.2%) [25,26,28,32,34,35,39,40,43,46]. Eight studies (25.8%) used a condition or diagnosis variable to guide their dispatch [20, 22, 23, 32, 36, 38, 40, 41, 52, 54]. ISS was not the only injury score used, the RTS and AIS were used eight times in total (24.8%) with four occurrences each (12.4%) in seven studies [23, 26–28, 46]. Blood pressure and heart rate were used fourteen times each (45.8%) across seven studies (22.5%) [25,28–30,40,42,53]. Respiratory Rate (RR) was only recorded in 6.4% (*n* = 2) of studies [26, 28]. Temporal measures included the data on the timings associated with the dispatch of PHCC assets. Unsurprisingly, the largest contributors to this group were mobile times (11/31(35.4%)) [8,9,20–22,32,38,39,42,44,50] and activation times (10/31(32.2%) [8,9,21,22,32,38,39,42,44,50]. On-scene times [8, 41, 50, 52, 54] and the entire prehospital phase times [36, 41, 50, 52, 53] are featured as variables in seven studies, occurring five times each (16.1%), with transport times occurring in four of the thirty-one studies (12.9%) [22,38,42,52]. Statistical measures were recorded in four of the 31 studies (12.9%) and grouped into six variables for data extraction. Over and under-triage rates were recorded in three studies each (9.6%) [8,26,46], sensitivity/specificity used twice (6.4%) [23,47] with positive/likelihood ratios [38] and PPV/NPV, [47] recorded one time each (3.2%). Demographic variables included time (6/31(19.4%)) [22,23,34,39,50,53], date (5/31(16.1%)) [23,34,39,50,53], location (3/31(9.6%)) [34,39,52] and co-morbidities 1/31(3.2%)) [22].

Outcome data was commonly associated with studies 20(50%) when a clinical prediction rule was derived, validated or assessed for impact. Fourteen (65%) of these tested a clinical prediction rule for internal validity [8, 9, 23, 24, 26–28, 30, 33–36, 46, 50] four (20%) for impact [25, 48, 51, 54] and three (15%) where the derivation of a rule occurred [43–45].

## Discussion

This review identified 39 studies, mostly retrospective and from high-income countries. Key findings included six main variable categories, frequent use of outcome and dispatch measures, and clinician involvement in over half of the dispatch models. Significant variation in systems and limited prospective research highlight ongoing gaps in evidence.

Most of the studies in this review are retrospective in their study design, with only one randomised control trial identified during the scoping review. This may reflect the challenges of conducting research in this area, relating to cost implications and ethical considerations. In one doctoral study, the patient and public involvement group vehemently opposed the idea of randomisation in potential research involving PHCC teams and cardiac arrest care [55]. In addition, relatively low call volumes and the disparate nature of the provision of services may also prove challenging in powering a study effectively and adequately to determine statistical significance. In this review alone, there is wide heterogeneity in dispatch staffing model, aetiology of calls attended and staffing models for PHCC teams, even amongst individual nations. Accounting for these differences in a large prospective study would require large cohorts to adequately power studies, adding further complexity and cost. In one epidemiological study, the rate of patients experiencing critical illness in Sweden was as low as five incidents per 10,000 person-years [56]. It would require significant time and investment to account for and control variables likely to change over a prolonged period. This may be why research designs like Delphi studies (which rely on expert consensus) and retrospective studies have been more prevalent in this review than designs like RCTs. They are comparatively cheaper and easier to conduct, which aligns well with the complexities and challenges of PHCC research.

All but one of the countries featured in the review took place in high-income countries (GNI per capita of $14,005) [57] (Fig. 3). South Africa was the only country classed as upper-middle income (GNI per capita of $4,516 to $14,005) [57]. While prehospital critical illness and injury are not diseases limited to the Western world, it is unsurprising that low- and middle-income countries are not heavily featured in this review, likely due to the extensive resources required for an effective PHCC system. Most studies in this review concentrated on dispatching HEMS-based teams, which constitute an expensive resource in the field, even for high-income countries. Furthermore, the prehospital phase represents a small portion of a critically ill or injured patient’s pathway, and there is also a need to care for these patients downstream of their prehospital treatment. PHCC systems require services such as trauma networks and larger tertiary hospitals with specialist care to achieve maximal outcomes. Unfortunately, these services remain beyond the reach of most Low and middle-income countries, making services like PHCC teams unsuitable for an adequate distribution of scarce resources. A more relevant question for this review may be why there is so little published data from the other eighty-six high-income countries, some of which have well-developed PHCC services and downstream care. And why, despite Delphi studies [58, 59] (one dating over a decade ago) highlighting the importance of research in this area, so little attention has been paid to PHCC dispatch. According to this review, the number of studies has remained relatively consistent over the last two decades, averaging two a year. Despite this, it seems some crucial questions have attempted to be answered.

Several studies attempted to look at the optimal models for dispatch staffing. In some studies, it wasn’t easy to ascertain who was dispatching the PHCC assets as most did not explicitly state who was involved. While small in number, a few studies attempted to answer the question. Only one study found that non-clinically trained staff, following a bespoke algorithm, could adequately dispatch PHCC assets [9]. However, in more studies, dispatch rates improved when clinicians managed dispatch within the control room [8, 24, 25, 48]. This effect improves according to experience and qualifications, with physicians embedded within HEMS teams showing better case identification and reduced cancellation rates over non-critical care-qualified paramedics [24, 25]. It is likely a combination of factors, including knowledge, experience, and other naturalistic decision-making methods like recognition-primed decision-making, play a large part in this [60]. No studies included in this review looked at the decision-making processes during PHCC dispatch and what role cognitive tools like heuristics and biases play when deciding to task specialist assets. However, following completion of this scoping review, Morton et al. (2025) investigated the decision-making involved with the dispatching of a physician paramedic critical care teams [61].

The advanced intervention variable was the most used variable within the outcome subgroup and is reasonable given that this would signify a successful dispatch. When this variable was applied to cancelled HEMS calls after arrival at the hospital, researchers found that HEMS was incorrectly stood down in a significant number of cases (20.6%, 32/155), representing a missed opportunity in these instances. One study looking at major trauma patients over three years in the North of England trauma network found 1202 cases where a PHCC-type intervention (and death < 24 h) was performed after arrival at the hospital with no involvement of an enhanced care team [62]. A similar study in Wales found 304 patients requiring tracheal intubation after arrival at the hospital [63]. Both studies found an improvement in mortality after treatment by an enhanced care team, representing a significant proportion of patients in these systems who have not received PHCC that may have benefited from doing so. The reasons for this remain unclear and could be related to several factors, including proximity to the hospital or unavailability of PHCC teams due to events like weather or maintenance of airframes. Whilst several of the studies included in this review have assessed the sensitivity and specificity of various triage tools, only one has looked at the broader prevalence of missed calls, and this was only in the cancelled cohort of patients attended by their service [28]. Thus, the sensitivity of tools, such as clinical prediction rules used for dispatch, may be considerably lower than what is reported when the missed opportunity for a PHCC-type intervention is taken into account in this broader cohort of patients who were not identified during the initial call process, but later required intervention. A limiting factor in assessing the accuracy of dispatch decisions may be limited by the ability to match outcomes across the entire patient cohort, which includes missed opportunities.

The changing distribution of demographics across Western countries could mean using the same clinical prediction rules for dispatch across all age groups is no longer accurate. Griggs et al. [46] found that in the traumatically injured > 65 years age group, PHCC-type interventions increased for secondary dispatches after assessment by the attending crew. This signifies a potential under-triage in their triggers for dispatch. They postulated that the mechanism of injury criteria are less sensitive in predicting the need for PHCC team attendance in low-energy trauma patients in this age group. Mechanism of injury was widely used across studies in this review that focused on trauma dispatch despite being a poor predictor for major trauma, especially when used in isolation [64, 65]. Combining Mechanism of injury with physiological parameters and/or other trauma outcome scoring may improve the predictability to acceptable levels [66, 67]. This finding echoes the results of a previous systematic review of HEMS dispatch in 2017 [31]. However, it remains inherently challenging to ascertain physiological variables during the receipt of the initial emergency call. It may account for why, in some instances, the criticality of the patient is not realised until after assessment by an initial attending ambulance crew and why MOI remains a proxy in their absence. Pushing this assessment closer to the point of call using novel technologies like GoodSam (Smartphone Activated Medics; www.goodsamapp.org), as seen in one study in the review [49] may allow a more subjective patient assessment. It can also provide dispatchers with objective measurements such as pulse and respiratory rates to better guide dispatches. Automatic crash recognition is another novel dispatch method that relies on burgeoning technologies to draw on additional data points to guide dispatch. However, with research confined to small feasibility studies and case reports within a single country [39,42], this remains an area for further exploration.

### Limitations

Due to the large number of studies identified, the initial screening was limited to title and abstract, which may have led to some missed papers. The initial search included terms of less common aetiologies PHCC teams encounter, such as CVA and maternity, resulting in over 20,000 results. It was felt this was excessive for a small team conducting a scoping review. In comparison, reviews on trauma generated about 1,200 results. It was determined that 4,804 results offered a manageable amount and sufficient terms to cast the net wide enough. Some information, such as crew composition, dispatch staffing models and systems, was not explicitly mentioned in the reviewed manuscripts. Potentially, there may have been some data capture issues in this method as it may not have been immediately evident from the articles what the crew mix was. In some instances, the research team could be assured that the country of origin only used physicians in their HEMS crew makeup (i.e. Japan) or solely Paramedic (i.e. Canada). Consequently, it appeared prudent to classify these items into their respective categories, following a reflexive approach commonly utilised in scoping reviews [68, 69]. The heterogeneity of professional terms and response vehicle nomenclature also led to some difficulty with data extraction in this category. Some studies explicitly stated that Rapid Response Vehicles or GEMS were also dispatched as part of P-HEMS or HEMS response. All HEMS or P-HEMS response categories likely use a mixture of rotary and ground assets due to weather limitations or practicality for responding to incidents close to airbases. However, these were not explicitly mentioned in the articles. There may also be an overlap in the subcategories of outcome variables. In all cases where ambiguity existed, terms were designated under consensus by PO and JH to improve readability. While they may seem arbitrary, they should not affect the presentation of individual variables. The numbers presented for dispatch arrangements are much lower than those mentioned in the dispatching staffing section; these were included in the analysis as a variable, for instance, where internal validation of a particular model was being assessed. In the remaining studies, the dispatch staffing model was mentioned explicitly in the study design or inferred from the research team’s current or previous working involvement with the described system at the time of data collection or publication.

## Conclusion

Whilst there is a broad spectrum of research in PHCC dispatch, it is limited in design to mainly retrospective studies, reviews and consensus statements. Whilst speculative, the reasons for this may be due to the difficulty of conducting empirical studies that are prospective in nature because of ethical, financial and statistical challenges faced by researchers in this field. Clinician involvement and the experience of these individuals appear essential to optimise the dispatch process. There is a paucity of information on the actual burden of missed opportunities in dispatch and why some critically ill or injured individuals fail to receive an adequate PHCC response. Understanding the causality of these mechanisms is vital to address this critical issue. It would require harmonisation across prehospital and hospital datasets to capture these patients adequately and allow better matching of dispatch and patient outcomes. Shifting the ability of dispatch staff to incorporate novel technologies to improve diagnostic capabilities at the point of call also seems worthy of exploration. This will provide objective and subjective measures to enable better decision-making earlier, reducing time to dispatch and improving outcomes.

## Supplementary Information

Below is the link to the electronic supplementary material.

Supplementary Material 1

Supplementary Material 2

## Data Availability

Full charting table is available on request.

## Abbreviations

AIS: Abbreviated injury score
ACN: Automatic crash notification
ECC: Emergency call centres
EMS: Emergency medical services
HEMS: Helicopter emergency medical services
GEMS: Ground emergency medical services
GCS: Glasgow come score
ISS: Injury severity score
ITU: Intensive care unit
NPV/PPV: Negative/positive predictive value
PHCC: Prehospital critical care
P- HEMS: Physician-staffed HEMS (P-HEMS)
RTS: Revised trauma score
