# Supplementary material for: Prehospital critical care dispatch: a scoping review (PHASE)
Source: Scand J Trauma Resusc Emerg Med. 2025 Aug 14;33:140. doi: 10.1186/s13049-025-01450-y (PMC12351921; doi:10.1186/s13049-025-01450-y)
Supplement: Supplementary file 1 — Supplementary Material 1 [file 13049_2025_1450_MOESM1_ESM.docx]

| *Summary of Characteristics for selected studies* | | | | | | | |
| --- | --- | --- | --- | --- | --- | --- | --- |
| Author, Year and reference number | Study design | Country in which the study was conducted | Type of PHCC asset being dispatched | Condition | Total number of participants | Aim of study | Conclusion |
| Berkeveld et al. 2021[28] | Retrospective observational | Netherlands | P-HEMS | Medical and Trauma | 18076 | Examines the current cancellation rate in trauma region over a six-year period. Cancellation reasons are also evaluated. | Increased cancellations for the 6-year period compared to 10-15 years ago. Paediatric incidents had higher cancellation rates. |
| Braude, Hutton and LaValley. 2005 [29] | Retrospective observational | United States | HEMS | Medical and Trauma | 30185 | Establish dispatch, lift-off and scene time benchmarks for emergency rotor-wing scene flights. | Significant delay in lifting during weather months. |
| Charrin et al. 2024 [30] | Cross sectional study | France | Mobile medical team (Physician Led) | Respiratory | 870 | Evaluate the impact of interrogated MMT dispatch compared to immediate dispatch at the time of the call on patient outcomes. | Immediate deployment of an MMT to patients in acute respiratory distress may result in decreased short to medium-term mortality compared to a delayed MMT dispatch. |
| Eaton et al. 2017 [31] | Service Evaluation | UK | HEMS, RRV | Medical and Trauma | 1171 (Dispatches) 734 (Attended scene) | Determine which dispatch criteria for ECTs are more efficient at targeting deployment to critically ill patients. | Within this service evaluation, broader call types were shown to be the most accurate dispatch criterion, and specific dispatch criteria (such as RTC entrapment) were shown to be the least accurate. |
| Eaton et al. 2018 [32] | Systematic review | UK | HEMS | Medical and Trauma | 14 Studies | Overview of the evidence for dispatch models of helicopter emergency medical services to critically ill or injured patients. | Standardised dispatch not suitable for all HEMS services. Physiological data is important in guiding decision making. |
| Edmunds et al. 2023 [33] | Retrospective observational | UK | HEMS, RRV | Medical and Trauma | 23030 (Dispatched) 8437 (Patient contact with HLIDD) | Define the dispatch criteria available at the time of the initial emergency call with the greatest HEMS utility. | Identified nine ‚AMPDS that UK EMS should consider to guide immediate HEMS dispatch. |
| Franschman et al. 2013 [34] | Retrospective observational | Netherlands | P-HEMS | Trauma | 334 | To evaluate whether this extended availability increased P-HEMS dispatch in patients with severe TBI. | P-HEMS is deployed more often for severe TBI with extracranial injuries. Dispatch rates are lower in urban areas despite similar ISS scores, and paramedic-led treatment is more likely to result in secondary transfers |
| Garner, Lee and Weatherall. 2012 [35] | Retrospective observational | Australia | HEMS | Trauma | 99 | Compare the screening process used by the HIRT team versus RLTC in children with severe trauma for case identification rates and transfer to PMTC | Physician-staffed HEMS crew screening and triage of emergency are more effective at identifying cases of severe paediatric trauma than a centralised screening system staffed by non- HEMS paramedics. |
| Garner et al. 2016 [36] | Retrospective observational | Australia | P-HEMS | Trauma | 197 | Evaluate the effect of this change in tasking systems on the Sydney paediatric trauma system. | Relying only on RLTC paramedics for case identification resulted in lower identification rates, longer prehospital times, and no significant change in direct transfers to the PTC. Removing PHEMS input worsened performance |
| Giannakopoulos et al. 2010 [37] | Retrospective observational | Netherlands | P-HEMS, RRV | Medical and Trauma | 501 (Dispatches) | Evaluate the cancellations of our MMT dispatches and to define the possible causes of this form of over triage. | MMT dispatches have rates of over triage. MMT is cancelled in almost 50% of all cases. |
| Giannakopoulos et al. 2011 [38] | Retrospective Observational | Netherlands | P-HEMS | Trauma (Blunt) | 708 | Assess if a maximum on-scene RTS (=12) can be used as a safe triage tool for HEMS cancellation. | RTS results in under triage (1 in every 6 cancellations) |
| Giannakopoulos et al. 2012 [39] | Retrospective observational | Netherlands | P-HEMS | Trauma | 606 (total dispatches) 420 (included in analysis) | Assessed the ability of the existing primary HEMS dispatch criteria to identify major trauma patients. | New cancellation triage model, can identify major trauma patients do not need HEMS over and under triage rates improved |
| Gries et al. 2014 [40] | Retrospective observational | Germany | P-HEMS, RRV | Medical Trauma | 2111 | Investigate rescue equipment planning, supply times and selection of the target clinic for joint operations of ground-based and air-based rescue equipment in Hesse | Evidence for the need to introduce algorithms, alarm sequences, disposition criteria, and nationwide integration of electronic support systems in central control centers |
| Griggs et al. 2021 [41] | Cohort study | UK | HEMS | Trauma | 1422 | Explore HEMS dispatch accuracy in older trauma patients by analysing HEMS specific interventions and disposition of this sub-group of trauma patients, stratified by dispatch type. | Many patients not eligible for immediate HEMS dispatch still require advanced interventions and tertiary care at major trauma centers. |
| Harmsen et al. 2017 [42] | Delphi | Netherlands | P-HEMS | Trauma | Round 1 83 Round 2 79 Round 3 71 | To elucidate factors of influence on prehospital triage and the identification of the severely injured patient. | The respondents report that prehospital communication needs to be unambiguous to improve trauma care. |
| Hewitt et al. 2020 [43] | Service Evaluation | UK | HEMS | Trauma | 2318 | This study sought to determine the temporal spatial distribution of trauma and if these needs are met by current services. | There is a significant difference between the current trauma care provision and the demand of major trauma incidents. |
| Kehoe et al. 2008 [44] | Retrospective observational | UK | P-HEMS | Trauma | 805 | This study was conducted to locally validate the immediate dispatch criteria. | In major trauma, some mechanism-based dispatch criteria reliably predict severe injury, physiological burden, resource use, and mortality. |
| Laatz. Welzel and Tyson. 2019 [45] | Delphi | South Africa | HEMS and GEMS | Medical and Trauma | 118 | Utilise expert opinions to reach consensus on HEMS call-out criteria. | Proposed an initial HEMS eligibility screening concept using expert opinion and literature-based dispatch criteria, requiring further research and validation. |
| Littlewood et al. 2010 [46] | Cross sectional study | UK | HEMS | Medical and Trauma | 16 services | Establish and compare the tasking criteria, dispatch arrangements and crew configuration for all helicopter ambulance services in the United Kingdom | Given the financial burden and physical risk of air ambulance use, there should be a more standardised approach to the tasking, dispatch and crew configuration of air ambulances in the UK. |
| Matsumoto et al. 2016 [47] | Non-randomised experimental study | Japan | HEMS | Trauma | 1 | Validate the feasibility of early HEMS dispatch via AACN. | Demonstrated that it is feasible to automatically alert and activate the HEMS via AACN. |
| McQueen et al. 2015 [16] | Systematic review | UK | Enhanced care assets' | Medical and Trauma | 16 Studies | Examine the evidence for primary and secondary models in the targeted deployment of ECT resources to patients with severe traumatic injuries | Lack of evidence supports primary dispatch models with dedicated criteria in optimising Enhanced Care Teams' deployment for severely injured pre-hospital trauma patients. |
| Motomura et al. 2020 [48] | Non-randomised experimental study | Japan | HEMS | Trauma | 4 | Assess the effectiveness of AACN in dispatching HEMS. | DCN leverages advanced sensor and communication technology to dispatch doctors to traffic accidents, potentially saving lives. |
| Munro et al. 2018 [18] | Non-randomised experimental study | UK | P-HEMS | Medical and Trauma | 4703 (2510 in period one and 2184 in period two) | Whether train non-clinical dispatchers are comparable to clinicians in identifying calls and dispatching HEMS | Non-clinical, HEMS-specific dispatch using a bespoke algorithm improved tasking accuracy, warranting further research for broader application. |
| Nolan et al. 2018 [49] | Retrospective observational | United States | HEMS | Trauma | 290 Total 102 cancelled 24 later transported to MTC | Ascertain the frequency of canceled HEMS scene calls that were later transferred to level 1 trauma centres. | Patients with canceled HEMS responses later transported to trauma centers face morbidity and mortality similar to those taken directly, highlighting significant under triage. |
| Oelrich et al. 2022 [50] | Retrospective observational | Denmark | RRV (Physician) | Medical and Trauma | 2370 (Assessed for eligibility) 1947 (included) 423 (Excluded) | Evaluate the type of patient groups involved in ambulance rendezvous with physician based RRV. | The high rate of unclear dispatch criteria highlights challenges in precise dispatch and resource use, necessitating a safe, rapid rendezvous procedure. |
| Otaguro et al. 2024 [51] | Case report | Japan | P-HEMS | Trauma | 1 | A case report in which the DCN effectively facilitated optimal care for a patient with severe trauma due to a single-vehicle accident. | DH request was shortened significantly due to the DCN. |
| Petrie et al. 2007 [52] | Retrospective observational | Canada | HEMS | Medical and Trauma | 578 (Identified) 563 (after exclusions removed) | Describe a unique mission acceptance/triage process in a single, provincially based, HEMS system. | A unique mission acceptance/triage process in based HEMS system is described. The rate of medically appropriate missions in this system is relatively high. |
| Ringburg et al. 2005 [53] | Retrospective observational | Netherlands | P-HEMS | Medical and Trauma | 6117 total (1148 primary +  38 secondary = 19%) | Analyse actual dispatch rates and assess the protocol adherence of the emergency dispatchers in Rotterdam regarding HMMT dispatch. | HEMS use in South-West Netherlands is inefficient and does not align with prehospital HMMT needs. Adhering to protocols could improve PDs sevenfold, ensuring better healthcare resource allocation. |
| Ringburg et al. 2009 [54] | Systematic review | Netherlands | HEMS | Trauma | 34 Studies | The purpose of this review was to provide an overview of HEMS dispatch criteria for patients with traumatic injuries described in the literature. | Only 5 studies described HEMS dispatch criteria validity. HEMS dispatch based on consciousness criteria seems promising. MOI and age-based criteria lack accuracy. |
| Samdal et al. 2021 [55] | Retrospective Observational | Norway | P-HEMS | Medical and Trauma | 19,028 (with primary dispatch) 2,506 (Incidents with P-HEMS dispatch). | Evaluate P-EMS availability, the underlying criteria for dispatch, dispatch accuracy and identify areas for improvement. | P-HEMS availability and dispatch accuracy in southeast Norway are suboptimal due to vague criteria and inconsistent prehospital data. Future criteria should prioritize P-EMS care. |
| Sinclair et al. 2018 [56] | Randomised controlled trial | UK | P-HEMS, RRV | Trauma | 454 PHCCT dispatches. 115 (Pre-TD) 339 (Post-TD). | Accuracy of the trauma desk tasking system to define major trauma patients and compare to the previous tasking model. | A PHCC clinician located in ambulance control can identify major trauma patients as early as possible and ensure appropriate resource tasking to those patients. |
| Skjærsethrseth et al. 2021 [57] | Delphi | Norway | HEMS | Medical and Trauma | 38 experts invited. | This study aimed to develop and implement QIs before establishing HEMS coordination and flight following by the regional EMCC in central Norway. | Using the Delphi method, we developed QIs for HEMS coordination and flight following with input from an experienced expert panel. Fourteen QIs achieved consensus. |
| TerAvest et al. 2019 [58] | Diagnostic test accuracy study | UK | P-HEMS | Medical and Trauma | 21 | Describe the acceptability and feasibility of using live video footage as a dispatch aid for HEMS dispatchers. | Live video from scenes is a feasible aid for HEMS dispatch. Further studies should assess its impact on accuracy and broader use in ambulance services. |
| Tiamfook-Morgan et al. 2008 [59] | Retrospective observational | United States | HEMS | Trauma | 100 | Assess one HEMS service compliance with dispatch guidelines and determine whether there were identifiable factors associated with out-of-guideline helicopter use | There is benefit to incorporating region based analysis into the utilization review process, because such analysis can assist in focusing ongoing educational efforts. |
| Wigman et al. 2011 [60] | Service evaluation | Netherlands | HEMS | Trauma | 77 (Identified) 12 (unable to be contacted) 55 | Overview of dispatch criteria related to trauma dispatch used by HEMS organisations in Europe, comparing countries and HEMS stations. | This overview demonstrates the lack of uniformity in the use of trauma-related dispatch criteria on a national and international level. |
| Wilmer et al. 2015 [17] | Service evaluation | UK | P-HEMS | Trauma | 2203 | Which dispatch methods employed by a doctor-led prehospital trauma service are most accurate and effective in identifying patients with serious injury. | MOI is a common but least accurate dispatch criterion. Remote methods like MOI and interrogation identify most serious injuries without the delays of crew scene assessment. |
| Wish and Davis. 2005 [61] | Service evaluation | United States | HEMS | Medical and Trauma | 86 (Usable) from a possible 240 AAMS operator members. | Survey of Association of Air Medical Services (AAMS) members regarding their auto launch practices and protocols. | About half of respondents use auto-launch, though protocols vary. It may reduce EMS response times, but more research is needed to refine dispatch criteria. |
| Yanagawa et al. 2023 [62] | Retrospective observational | Japan | P-HEMS | Medical and Trauma | 28,357 patient records were analyzed (key  words group, n = 13,861; control group, n = 14,496) | Assess whether key words from emergency call can assist in dispatching doctor helicopter. | This is the first report identifying the keywords method as a survival factor for DH-evacuated patients via JDRS. Firefighting command should adopt this for HEMS use. |
| Zhang et al. 2022 [63] | Cohort study | Canada | HEMS | Trauma | 3232 (total calls) 1,465 canceled calls. However, 184 of those 1,117 (16.5%) were later transferred  to an LTH | Reasons for cancellations of HEMS trauma scene requests and rate of secondary transfer to MTC. | Most cancellations were due to patients not meeting trauma bypass criteria, but 16.5% were later sent to trauma centers. Education, adherence, and criteria updates are needed. |
