# Supplementary material for: Prehospital critical care dispatch: a scoping review (PHASE)
Source: Scand J Trauma Resusc Emerg Med. 2025 Aug 14;33:140. doi: 10.1186/s13049-025-01450-y (PMC12351921; doi:10.1186/s13049-025-01450-y)
Supplement: Supplementary file 2 — Supplementary Material 2 [file 13049_2025_1450_MOESM2_ESM.docx]

| Search Strategy | |
| --- | --- |
| 1 | Prehospital.mp |
| 2 | Prehospital critical care.mp |
| 3 | Advanced life support.mp |
| 4 | Out of hospital.mp |
| 5 | Helicopter emergency medical service.mp |
| 6 | HEMS.mp |
| 7 | Aeromedical.mp |
| 8 | Helicopter.mp |
| 9 | Air ambulance.mp |
| 10 | 1 OR 2 OR 3 OR 4 OR 5 OR 6 OR 7 OR 8 OR 9 |
| 11 | Trauma.mp |
| 12 | Traumatic brain injury.mp |
| 13 | Traumatic injury.mp |
| 14 | 11 or 12 or 13 |
| 15 | Cardiac arrest.mp |
| 16 | Arrythmias.mp |
| 17 | Sepsis.mp |
| 18 | Overdose.mp |

| 19 | 15 OR 16 OR 17 OR 18 |
| --- | --- |
| 20 | Dispatch.mp |
| 21 | Criteria based dispatch.mp |
| 22 | Allocation.mp |
| 23 | Ambulance operation centre.mp |
| 24 | Communication.mp |
| 25 | Machine learning.mp |
| 26 | Artificial intelligence.mp |
| 27 | Automatic crash recognition.mp |
| 28 | Wearables.mp |
| 29 | 21 OR 22 OR 23 OR 24 OR 25 OR 26 OR 27 OR 28 |
| 30 | Emergency Medical Dispatch.mp‘ |
| 31 | Advanced Medical Priority Dispatch System |
| 32 | Emergency Medical Service Communication Systems |
| 33 | 31 OR 32 OR 33 |
| 34 | 10 AND 14 AND 19 AND 29 AND 33 |
